# PPA-GCN: A Efficient GCN Framework for Prokaryotic Pathways Assignment

**DOI:** 10.3389/fgene.2022.839453

**Published:** 2022-04-04

**Authors:** Yuntao Lu, Qi Li, Tao Li

**Affiliations:** ^1^ Key Laboratory of Freshwater Ecology and Biotechnology, Institute of Hydrobiology, Chinese Academy of Sciences, Wuhan, China; ^2^ College of Advanced Agricultural Sciences, University of Chinese Academy of Sciences, Beijing, China

**Keywords:** graph convolution network, prokaryotic genome, metabolic pathway, deep learning, self supervised

## Abstract

With the rapid development of sequencing technology, completed genomes of microbes have explosively emerged. For a newly sequenced prokaryotic genome, gene functional annotation and metabolism pathway assignment are important foundations for all subsequent research work. However, the assignment rate for gene metabolism pathways is lower than 48% on the whole. It is even lower for newly sequenced prokaryotic genomes, which has become a bottleneck for subsequent research. Thus, the development of a high-precision metabolic pathway assignment framework is urgently needed. Here, we developed PPA-GCN, a prokaryotic pathways assignment framework based on graph convolutional network, to assist functional pathway assignments using KEGG information and genomic characteristics. In the framework, genomic gene synteny information was used to construct a network, and ideas of self-supervised learning were inspired to enhance the framework’s learning ability. Our framework is applicable to the genera of microbe with sufficient whole genome sequences. To evaluate the assignment rate, genomes from three different genera (*Flavobacterium* (65 genomes) and *Pseudomonas* (100 genomes), *Staphylococcus* (500 genomes)) were used. The initial functional pathway assignment rate of the three test genera were 27.7% (*Flavobacterium*), 49.5% (*Pseudomonas*) and 30.1% (*Staphylococcus*). PPA-GCN achieved excellence performance of 84.8% (*Flavobacterium*), 77.0% (*Pseudomonas*) and 71.0% (*Staphylococcus*) for assignment rate. At the same time, PPA-GCN was proved to have strong fault tolerance. The framework provides novel insights into assignment for metabolism pathways and is likely to inform future deep learning applications for interpreting functional annotations and extends to all prokaryotic genera with sufficient genomes.

## Introduction

With the rapid development of sequencing technology, the number of newly released prokaryotic genomes has exploded, providing an important foundation for subsequent research work (Doerks et al., 2004). Functional annotation and pathway assignment are important components of understanding the details of metabolism. Accordingly, a series of reference genome databases and functional annotation platforms have been developed (Benson et al., 2012; Federhen, 2012; Keegan et al., 2016; Chen et al., 2019; Bazgir et al., 2020). The Kyoto Encyclopedia of Genes and Genomes (KEGG) is one of the most widely used and reliable functional platforms, and it provides three annotation software tools, namely, BlastKOALA, GhostKOALA, and KofamKOALA, for functional annotation (Suzuki et al*.*, 2014; Kanehisa et al., 2016a; Kanehisa et al., 2016b; Aramaki et al*.*, 2020). Currently, only 48% of the protein sequences are assigned to pathways in the KEGG GENES database (Aramaki et al*.*, 2020). It is even lower for newly sequenced prokaryotic genomes, which has become a bottleneck for subsequent research (Suzuki et al*.*, 2014). Thus, the development of a high-precision metabolic pathway assignment framework is urgently needed.

Here, we propose PPA-GCN, a framework based on graph convolutional network (GCN) that uses genomic gene synteny information within specific genus, from which the graph topological pattern and gene node characteristics can be learned, to disseminate node attributes in the network and provide assistance to the assignment of metabolic pathways. Synteny is defined as two or more pairs of homologous genes occupying the same chromosomal segment, where homologous loci are defined based on the similarity of function of the products of the corresponding genes (Nadeau and Taylor, 1984). Analyzing synteny can provide insight regarding the evolution and function of genes (Zhang et al., 2016). As an inherent biological attribute, bacteria of different genera have different synteny patterns. In general, bacterial genomes have two different pan-genome types. The pan-genome refers to all genes detected in a whole group of genomes (Wang L. et al., 2020). Some prokaryotes have genomes with highly conserved gene content (closed pan-genomes), while others are more flexible (open pan-genomes). Since the concept of a “pan-genome” was first proposed in 2005, pan-genome analysis has revealed the diversity and evolution of bacterial genomes (Tettelin et al., 2005). In present, there is currently no deep learning framework for direct assignment of functional pathways against KEGG database. To evaluate PPA-GCN, genome datasets of three different genus were used, and on all of them, the proposed framework had achieved excellent performance. PPA-GCN enables novel insights into assignment for functional pathways and is likely to inform future deep learning applications for interpreting functional annotations.

## Related Work

The study of gene location in the genome is one of the classic fields of genetics (Rogozin et al*.,* 2004). In prokaryotes, genes encoding functional linked proteins are usually organized into gene clusters (Shmakov et al.*,* 2019). There were methods assign protein function using neighborhood properties (Saha et al.*,* 2012; Jun et al., 2017; Saha et al*.*, 2018). It has been shown that the neighborhood milieu of genes in a network can assist in predicting the probable function of a gene for which no function is known (Hao et al., 2012). However, there is almost no method to assign KEGG pathways using gene neighborhood information.

In recent years, deep learning has been widely used in the field of life science, for example, for identifying and interpreting the contextual features of transcription factors (Zheng et al., 2021), generating functional protein sequences (Repecka et al., 2021), and identifying cell types (Lukassen et al*.*, 2020; Wang M. et al., 2020). At present, the applications of graph neural networks in the medical and biology fields show strong representation and integration capabilities (Wu et al*.*, 2020), including neuroimage analysis (Zhang et al., 2018), disease gene identification (Li et al., 2019; Schulte-Sasse et al., 2021), drug combination synergy prediction (Zitnik et al., 2018; Jiang et al*.*, 2020; Manoochehri and Nourani, 2020), discovery of disease pathways (Agrawal et al*.*, 2018), prediction of tissue cell function (Zitnik et al., 2017), pseudogene function prediction (Fan and Zhang, 2020), conducting taxonomic classification for phage contigs (Shang et al., 2021) and identifying missing protein–phenotype associations (Liu et al., 2021). The graph convolutional network (GCN) is a type of graph neural network that can learn the structure of a graph. This network model was originally proposed for semi-supervised classification (Kipf et al., 2016). A GCN model can extensively integrate graph topological features and node information by defining each node as a computational graph and using neural networks to integrate neighbor node information.

## Materials and Methods

### Problem Statement

Given an undirected graph G = (V_tr_, V_te_, E), where V_tr_ is the set of nodes that assigned function pathway, V_te_ is the set of nodes that unassigned function pathway, V = {V_tr_, V_te_}. E is the set of edges and the edge represents two genes belonging to different nodes are connected in the genome. A label set L = {l_1_, l_2_...l_k_} is formed according to the KEGG secondary class. The relationship between the node set and the label set is represented by a matrix Y_NxK_. Y_ij_ = 1, if there is a gene in node i has assigned to label j. Our goal is to assign the possible pathway labels to those nodes that have no labels.

### Framework

**FIGURE 1:**
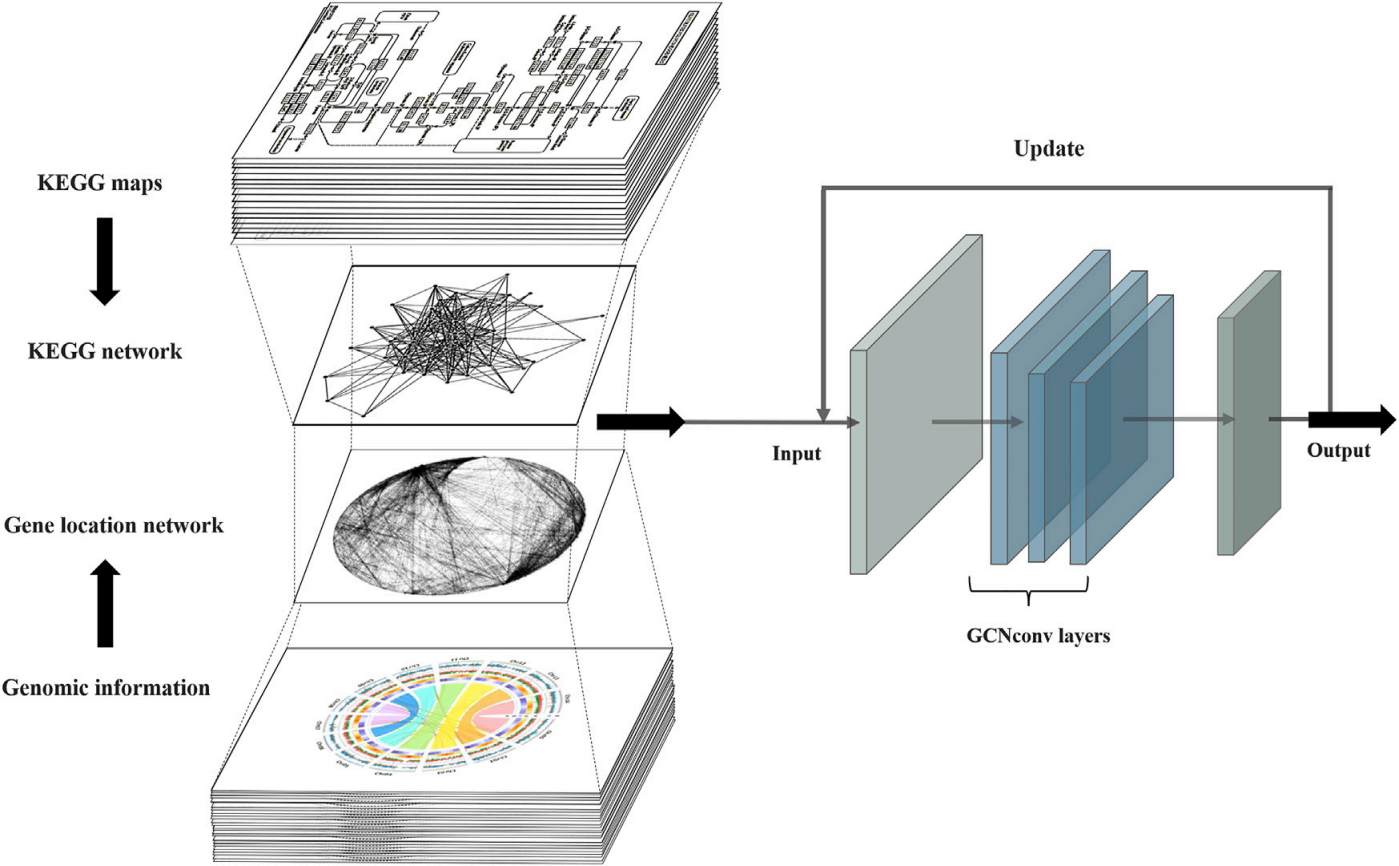
PPA-GCN architecture. The input to the framework is the metabolic pathway network extracted from the KEGG metabolic pathways and the gene synteny network composed of the prokaryotic genomes. The graph convolutional layer attempts to construct a mapping relationship between the two input networks and iteratively uses the training results to update the input inspired by self-supervised learning until a steady state is reached and the final assignment output is obtained.

PPA-GCN is a deep learning framework based on a graph convolutional model **(** Figure 1). Gene synteny information from the selected genome is used to construct edges in a network, while genes sharing high sequence similarity and cover ratio are grouped into nodes. All node and edge information are used to construct the gene synteny network. PPA-GCN applies a three-layer graph convolutional architecture. Input features include node encoding, node scale and adjacency probability matrix. The KEGG metabolic pathway information of the secondary class is used as the node labels for initial training. Improve performance with inspiration from self-supervised learning. The final outputs are ranked in accordance with the stability of the assignment during the training process.

### Graph Construction

#### Node Construction

Blast (Altschul et al., 1990) was used to compare the sequence similarity of all genome genes in one genus. In order to quickly and strictly find the similar genes, we directly adopted the reciprocal best hits comparison and controlled the identities and cover ratios to 65%. Taking *Flavobacterium* as an example, a total of 16,830 orthologs were obtained using OrthoFinder 2.0 (Emms and Kelly, 2019), and 51,247 nodes were obtained using our method, of which 50,998 nodes contained only one orthologs (99.5%). Therefore, our method is stricter than directly using orthologs. Node2vec algorithm (Grover and Leskovec, 2016) was used to generate graph embeddings for each node.

#### Edge Construction

**FIGURE 2:**
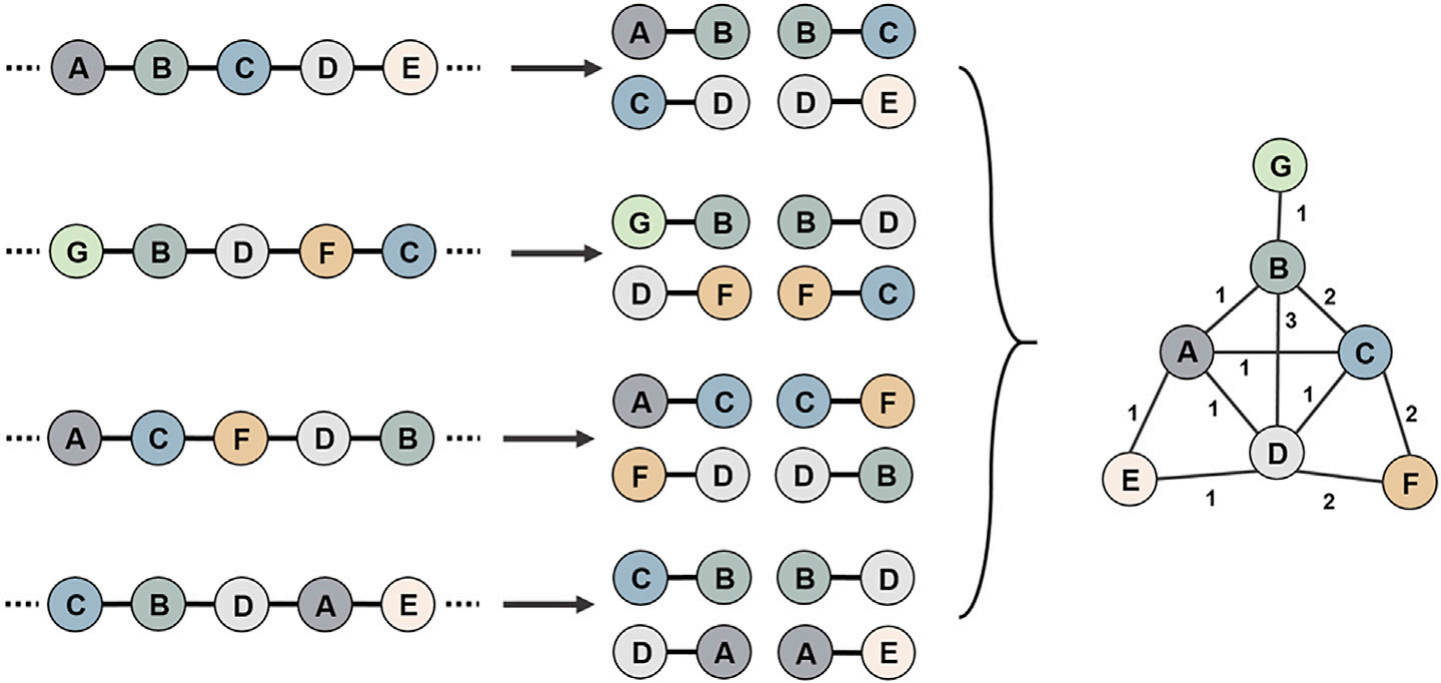
Schematic diagram of the use of multiple genomes to construct a gene synteny network. First, all genomic genes are compared for sequence similarity, and genes that share high reciprocal similarity and cover ratios are assigned the same node id. Then, positional relationship pairs between two genes from each genome were constructed. Finally, all gene position relationship pairs are connected into a gene synteny network.

Positional relationship pairs between two genes from each genome were constructed using the data of coding DNA sequence (CDS) (Figure 2). Through the correspondence between genes and nodes, all positional pairs were connected into a single gene synteny network, in which there could be more than one connection between two nodes. The adjacency matrix was constructed in accordance with the number of connections between nodes.

### Construction of the Adjacency Probability Matrix

The adjacency probability is defined as the probability that two nodes form a certain number of connections in the network. First, the degree of each node in the gene synteny network (the number of connections by which a node is directly connected to surrounding nodes) was calculated. Then, the probability *P*_*i*_ that an edge is connected to a specific node *i* was calculated. Finally, the probability that there are *k* edges between node *i* and node *j* was defined as:

$$P_{i}=\;\frac{degree\left(i\right)}{\sum_{n=1}^{N}degree\left(n\right)}\;$$ (1)

$$P_{ij}=C_{degree\left(i\right)\;}^{k}P_{j}^{k}{\left(1-P_{j}\right)}^{degree\left(i\right)-k}$$ (2)

where *N* is the total number of nodes in the gene synteny graph and *degree(i)* is the degree of node *i*, *C* is the combination symbol.

After the adjacency probabilities of all nodes had been formed into an N*N adjacency probability matrix, because there are no connections between most nodes, the node2vec algorithm was used to densify the adjacency probability matrix.

### The GCN Model

#### Framework Architecture

Given an undirected graph with node feature matrix *X* and adjacency matrix *A*, the graph convolution operation (Kipf et al*.*, 2016) is defined as:

$$H=\;\sigma \left(D^{\frac{1}{2}}\hat{A}D^{-\frac{1}{2}}XW\right)$$ (3)

$$\hat{A}=A+I,\;\;\;D_{ii}=\;\underset{j}{\sum }\hat{A_{ij}}$$ (4)

where *I* is the identity matrix, *W* is the matrix of trainable weights in the neural network, *X* is the feature matrix before the update, *H* is the feature matrix after the update, and *σ* is the activation function (ReLU). The graph convolution operation iteratively calculates the weighted average of the node attributes of the neighbors of the current node to obtain the new feature matrix of the node. In this framework, the features of unlabeled nodes (nodes without assigned functional pathways) and the features of nearby labeled nodes (nodes with assigned functional pathways) are mixed to be propagated through the synteny network diagram. If two nodes have the same neighbor structure and neighbor features, their embedded feature matrix *H* will be exactly the same.

Python’s PyTorch Geometric Module was used to implement PPA-GCN. Multiple graph convolutional layers can be stacked to enable learning on a larger domain structure. After testing, a three-layer stack was found to perform the best. The two-class cross entropy was used as the loss function because of the multilabel nature of the problem.

#### Self-Supervised Learning Inspiration

The original input was fed into the framework, and 50 epochs of random sampling verification training were performed with the test set. The nodes with an average cross-validation accuracy rate of less than 30% are removed from the training set, and nodes and labels with a assignment stability of 90% in the test set (that is, the same label is assigned more than 45 times) are added to the training set. After many iterations, when the number of nodes in the training set reached more than 90% of the total number of nodes in the gene synteny network, the training was considered to have reached a stable state, and the final assignment results were output.

### Topological Analysis

#### Degree and Degree Distribution

The degree is defined as the number of all edge connections of a node in a graph, describing the first-order connection degree of the node. The degree distribution is an overall description of the nodes in a network, that is, the probability distribution or statistical distribution of the node degrees.

#### Clustering Coefficient

The clustering coefficient is used to describe the degree of clumping among the vertices of a network. Specifically, it is the degree of interconnection among the adjacent nodes of a node, describing the second-order connection degree of the node. For node *i* with degree *k*_*i*_, the local clustering coefficient is defined as:

$$C_{i}=\;\frac{2L_{i}}{k_{i}\left(k_{i}-1\right)}\;$$ (5)

where *L*_*i*_ is the number of connections among the *k*_*i*_ neighbors of node *i*. The overall aggregation coefficient of the network is characterized as the average value of the aggregation coefficients of all nodes.

## Result

### Data

All training genomes were downloaded from the National Center for Biotechnology Information (NCBI) database in June 2021 (https://www.ncbi.nlm.nih.gov/genome/browse#!/overview/). The datasets include *Flavobacterium* (Gram-negative, 65 genomes), *Pseudomonas* (Gram-negative, 100 genomes) and *Staphylococcus* (Gram-positive, 500 genomes). *Staphylococcus* has a closed pan-genome. The 500 genomes selected for this study contain 1,332,382 genes grouped into a gene synteny network of 10,074 nodes. *Flavobacterium* and *Pseudomonas* have open pan-genomes. The 65 *Flavobacterium* genomes and 100 *Pseudomonas* genomes selected for this study contain 243,834 and 550,752 genes grouped into 51,247 and 79,941 nodes, respectively.

KEGG internal annotation tool KofamKOALA (version 100.0, updated October 1, 2021) was used to assign genes to functional pathways. The pathway labels belonging to the global and overview maps category were removed. *Staphylococcus* had 400,478 genes (1,324 nodes) assigned to metabolic pathways, *Flavobacterium* had 67,529 genes (3,694 nodes) assigned to metabolic pathways, and *Pseudomonas* had 272,388 genes (12,429 nodes) assigned to metabolic pathways (Supplementary Table S1). The original assignment rates for the three genera were 7.2% (*Flavobacterium*), 15.5% (*Pseudomonas*) and 13.1% (*Staphylococcus*).

In order to verify the performance of the model, the new genome data of the three genera were downloaded from the National Center for Biotechnology Information (NCBI) database in October 2021 (newly released genomes were downloaded first). The datasets include *Flavobacterium* (30 genomes), *Pseudomonas* (50 genomes) and *Staphylococcus* (200 genomes).

### Evaluation Metrics

Pathway label assignment is essentially a multilabel classification problem. Hence, some commonly used evaluation indicators for binary classification problems are not suitable for PPA-GCN. We use six indicators to measure the effectiveness of the framework:

#### Prediction Rate of Assignment

PRA is the accuracy at the node level and is defined as the proportion of genes with at least one label assigned correctly.

#### Total Label Prediction Rate

The TLPR is the accuracy at the label level and is defined as the number of correctly assigned labels divided by the total number of labels.

#### Weighted Prediction Rate of Assignment

When a label is predicted for a node, we assign weights in accordance with the assignment probability, sum the WPRA of each label of a node to obtain the *WPRA* of that node, and divide by the total number of nodes to obtain the overall WPRA:

$$w_{prediction}=\;\frac{1}{N}\underset{k\in T_{i}}{\sum }\frac{2\left(I+1-k\right)}{I\;\left(I+1\right)}$$ (6)

where *N* is the total number of nodes, *I* is the number of labels for node *i*, *T*_*i*_ is the order of the correct label probabilities assigned for node *i* (from large to small), and *k* is the k-th ranked probability label that was assigned correctly.

#### Kappa Coefficient

The kappa coefficient is often used for testing consistency, that is, whether the assignment effect of the model is consistent with the actual classification effect. Its value is between -1 and 1. When the value is greater than 0.6, it is considered substantial, and when it is greater than 0.8, it is considered almost perfect. The calculation of the kappa coefficient is based on the confusion matrix:

$$kappa=\;\frac{p_{0}-p_{e}}{1-p_{e}}$$ (7)

$$p_{0}=\;\frac{\sum_{i}M_{ii}}{\sum_{ij}M_{ij}},\;\;p_{e}=\;\frac{\sum_{i}M_{i.}M_{.i}}{{\left(\sum_{ij}M_{ij}\right)}^{2}}$$ (8)

where *M* is the confusion matrix of the assignment results.

#### Hamming Distance

The Hamming distance is measure of the distance between the assigned and real labels, with a value between 0 and 1. A distance of 0 means that the assigned results are exactly the same as the real results, and a distance of 1 means that the model’s results are completely opposite to the desired results. This indicator is calculated as the number of erroneously assigned labels divided by the total number of labels.

#### Jaccard Similarity Coefficient

This coefficient is an indicator for comparing the similarity of two finite sets, defined as the size of the intersection of two label sets (the true label set and the assigned label set) divided by the size of the union. When this coefficient is 1, the assigned results are completely consistent with the actual situation; when the coefficient is 0, the assigned results are completely inconsistent with the actual situation.

### Results of Experiments

#### Results of Cross-Validation

**FIGURE 3:**
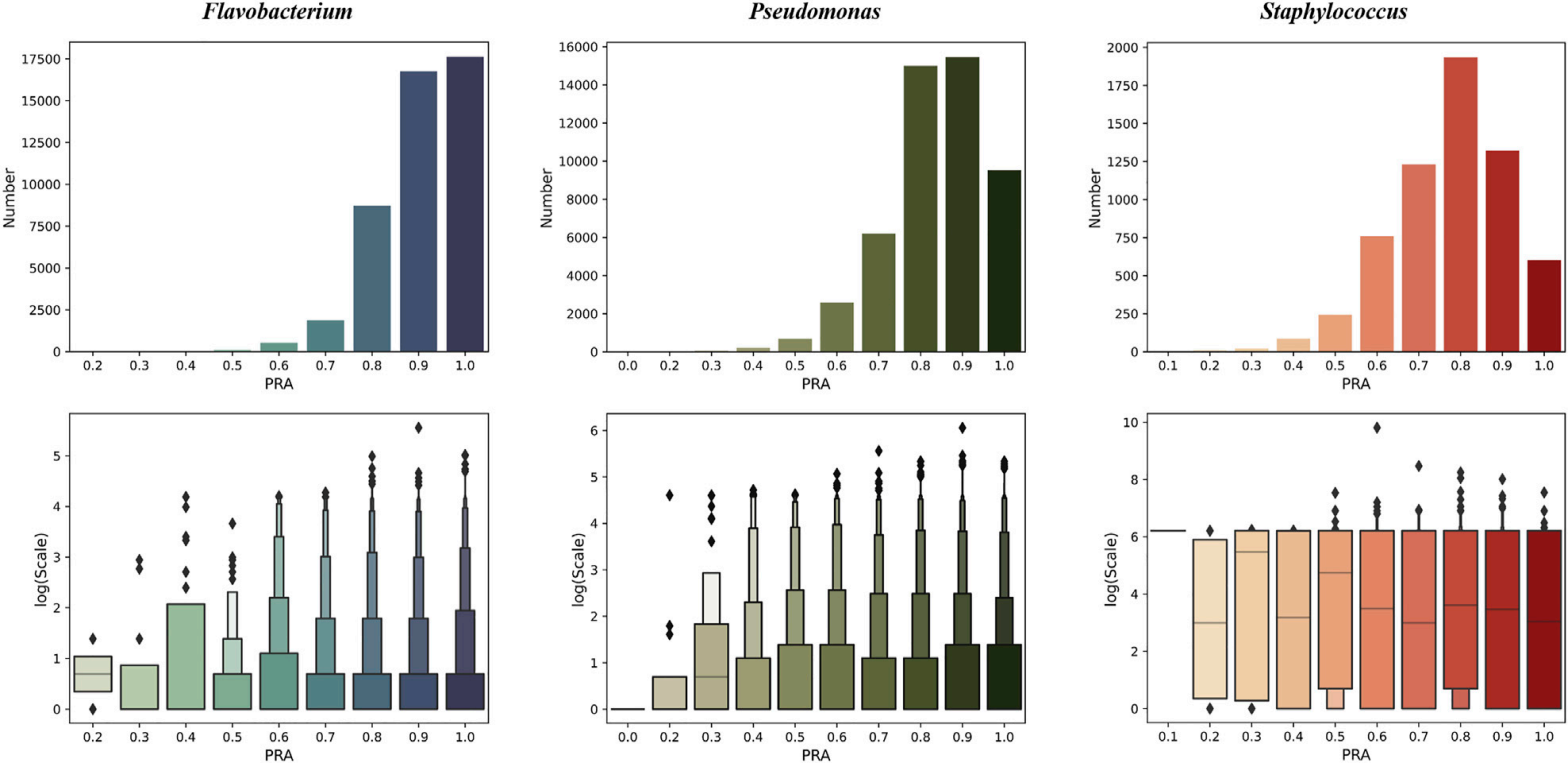
The performance of PPA-GCN on three genera (in terms of the PRA) and the node scale distribution of the node set at each PRA level (10% as one level). From left to right are *Flavobacterium, Pseudomonas,* and *Staphylococcus*.

We tested PPA-GCN with 5-fold cross-validation on three data sets. PPA-GCN achieved prediction rates of assignment (PRAs) of 84.8% (*Flavobacterium*), 77.0% (*Pseudomonas*) and 71.0% (*Staphylococcus*) on the three prokaryotic bacterial genera (Figure 3). According to the evaluation index results (Table 1), PPA-GCN is well adapted to all three genera.

**TABLE 1:** Performance under 5-fold cross-validation for the three genera.

Species	PRA	TLPR	WPRA	KC	HD	JS
*Flavobacterium*	0.848	0.846	0.829	0.842	0.008	0.751
*Pseudomonas*	0.770	0.728	0.736	0.721	0.014	0.609
*Staphylococcus*	0.710	0.691	0.698	0.689	0.008	0.651

In addition, we compared PPA-GCN with five other machine learning methods. deepNF (Gligorijevic et al., 2018), Mashup (Cho et al., 2016) and Pseudo2GO (Fan and Zhang, 2020) are three deep learning methods that use graph information for function prediction. Support vector machines (SVM) and deep neural networks (DNN) are two machine learning models that are not based on graph information. Using the *Staphylococcus* genome as the test data set, all methods use the same features in PPA-GCN as input, and use 5-fold cross-validation to test performance. The results (Table 2) show that, PPA-GCN achieves the best performance among all indicators.

**TABLE 2:** Performance comparison under 5-fold cross-validation

Methods	PRA	TLPR	WPRA	KC	HD	JS
deepNF	0.562	0.365	0.339	0.511	0.273	0.379
Mashup	0.562	0.446	0.479	0.529	0.108	0.450
Pseudo2GO	0.578	0.470	0.466	0.513	0.051	0.433
SVM	0.483	0.304	0.319	0.506	0.118	0.414
DNN	0.402	0.365	0.339	0.501	0.063	0.429
PPA-GCN (without self-supervised learning)	0.607	0.570	0.539	0.522	0.034	0.402
PPA-GCN	0.710	0.691	0.698	0.689	0.008	0.651

#### Results of Test

In order to evaluate the adaptability of PPA-GCN to new data, the genes of the new genome were classified into network nodes. The test set node of the newly assigned functional path label in the network was used as the evaluation object, and the difference between the assigned output label and the real label is directly compared. The results are shown in Table 3, which proves that the results of PPA-GCN is reliable.

**TABLE 3:** Performance under the new data set.

Metrics	*Flavobacterium*	*Pseudomonas*	*Staphylococcus*
PRA	0.637	0.613	0.798
TLPR	0.606	0.538	0.723

#### Fault Tolerance Evaluation

**FIGURE 4:**
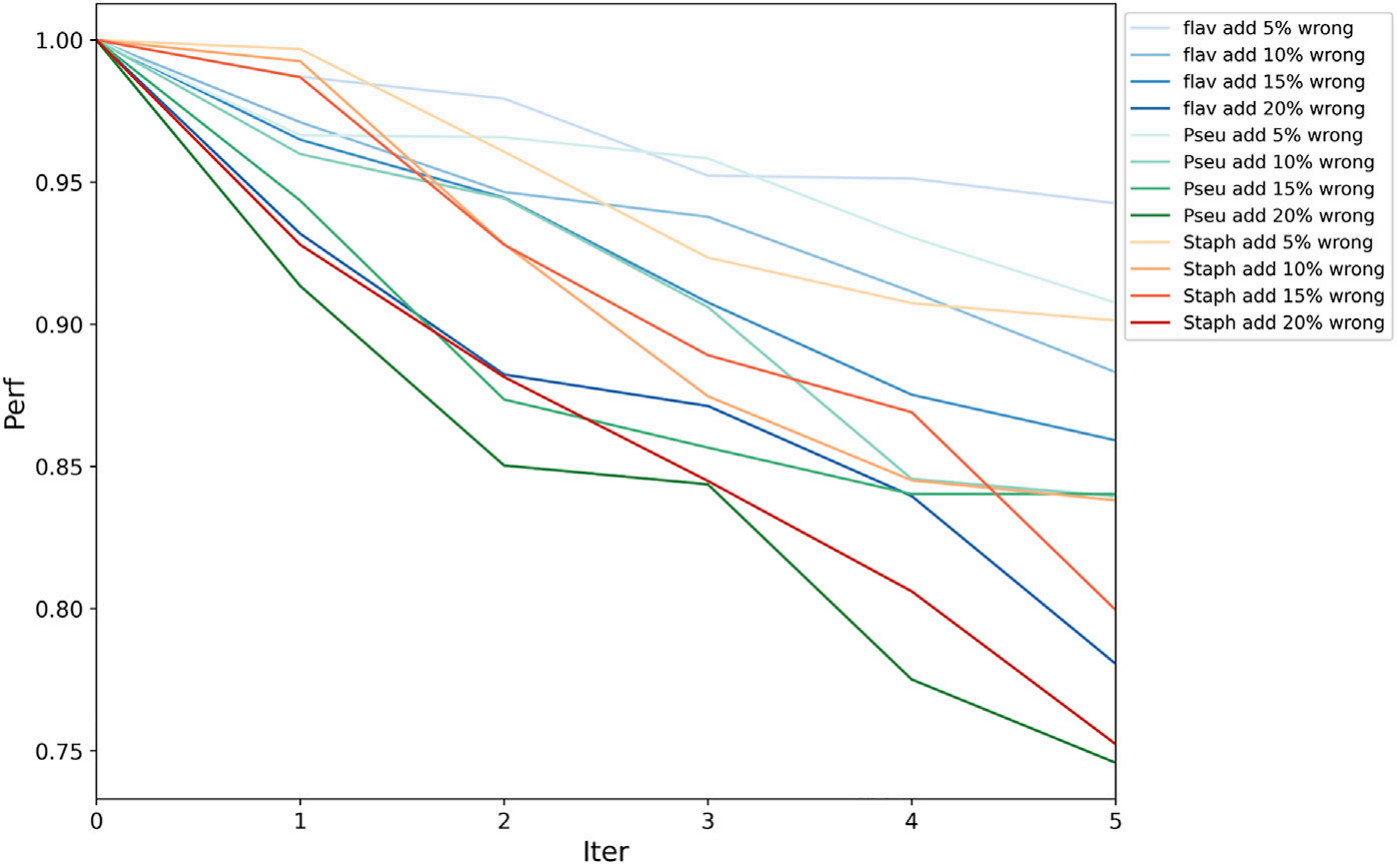
Framework fault tolerance evaluation. On the three datasets, the performance was tested with the accumulation of 5–20% incorrectly labeled data in each epoch; the horizontal axis is the number of iteration, and the vertical axis is the performance indicator (current PRA/original PRA). This result shows that PPA-GCN has strong fault tolerance.

Because functional pathway assignment for bacterial genomes is still in the development stage, there will inevitably be some false pathway labels on the bacterial genes. Hence, we needed to test the fault tolerance of PPA-GCN. All assigned labels were assumed to be correct. In each epoch of training, some unlabeled nodes were given random labels to also participate in the training process. Two sets of experiments were conducted. In one, a certain percentage (5–20%) of incorrectly labeled samples were added in each epoch independently, and in the other, incorrectly labeled samples were added accumulatively. The PRA without the addition of incorrect labels was taken as the standard, and the PRA after the addition of incorrect labels was divided by the standard PRA to serve as the performance indicator. The results (Figure 4, Supplementary Table S2) show that PPA-GCN can still maintain more than 75% performance with the addition of incorrect labels at a rate of up to 100% (that is, the incorrectly labeled samples compose up to 50% of the training set). Because the distribution of wrong labels is random, and the distribution of correct labels is ordered, the influence of correct labels on the training results is greater than that of wrong labels, which enhances the fault tolerance of the framework. With an increasing proportion of incorrect labels, the efficiency of the framework did not drop sharply. This result shows that PPA-GCN has strong fault tolerance.

#### Feature Importance Test

**FIGURE 5:**
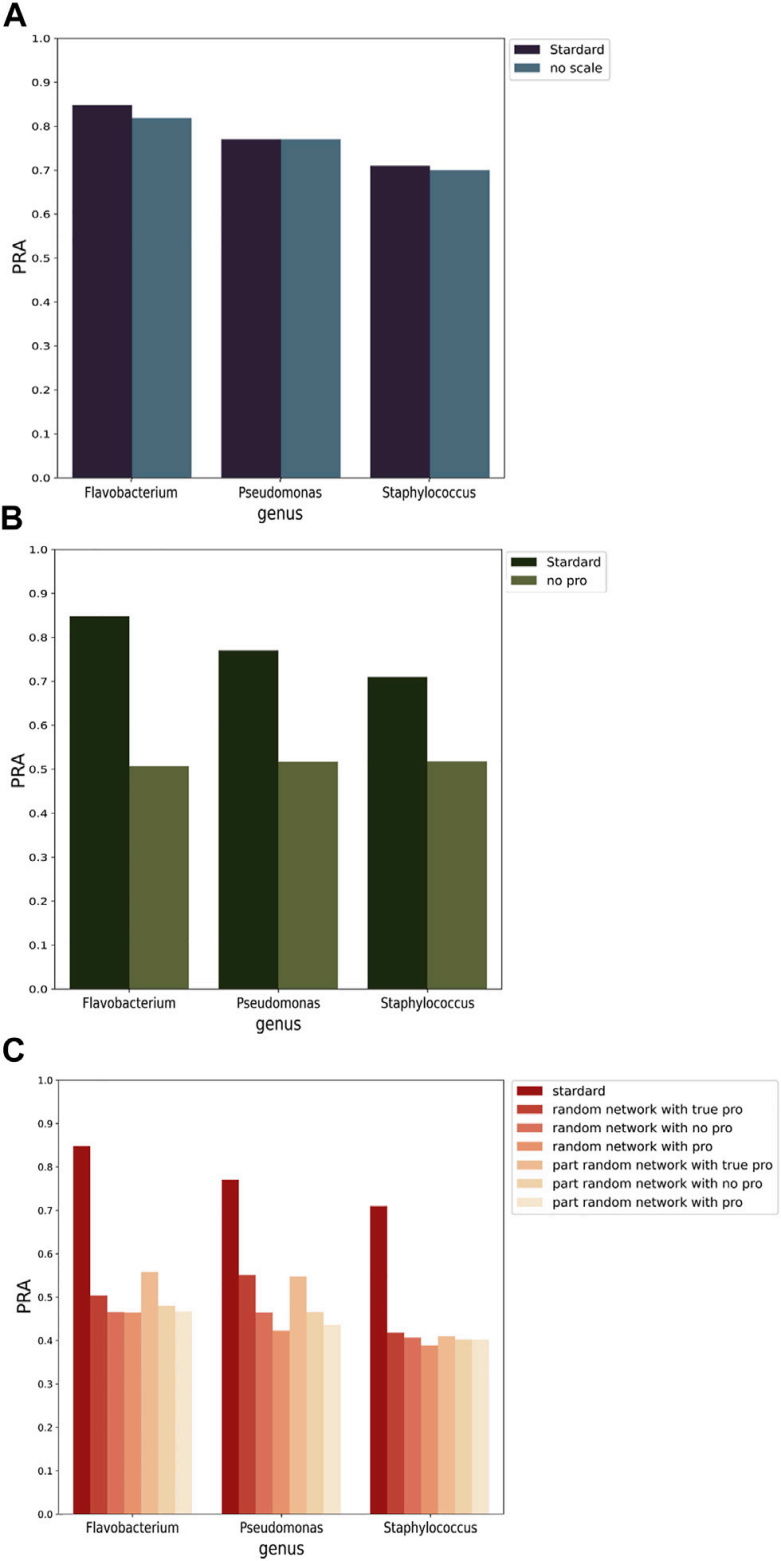
**(A)** Feature importance assessment of the node scale. Comparison of performance changes before (standard) and after removing node scale (no scale). **(B)** Feature importance evaluation of the probability adjacency matrix. Comparison of performance changes before (standard) and after removing probability adjacency matrix (no pro). **(C)** Feature importance assessment of the gene synteny network. The horizontal axis represents standard training and training using random networks generated with three strategies: not including any adjacency probability matrix (with no pro), including the adjacency probability matrix of the newly generated network (with pro), and including the adjacency probability matrix of the real network (with true pro). The three graphs all use the prediction rate of assignment (PRA) as the evaluation index. The results show that node scale, adjacency probability matrix and network are very important features of PPA-GCN.

A graph neural network can achieve excellent prediction accuracy, but it is difficult to give practical meaning to features. To evaluate the importance of the selected features, the PRAs before and after feature removal were compared (Figure 5, Supplementary Table S3). There are three important features in the PPA-GCN input: the node scale, the adjacency probability matrix and the gene synteny network.

The node scale is defined as the number of genes grouped into one node. The node scale was selected as an input feature because it can reflect the characteristics of a group of genomes. *Staphylococcus* has a closed pan-genome with an average node scale of 132.3, that is, an average of approximately 132 genes grouped into one node. *Flavobacterium* and *Pseudomonas* have open pan-genomes with average node scales of only 4.8 and 6.9, respectively. The node scale was one of the major observed differences between the labeled (training set) and unlabeled (test set) node sets in the gene synteny network. PPA-GCN showed no significant difference in performance when the node scale information was removed from the input (Figure 5A). The node scale has no effect on framework training, and this is beneficial for the applicability of the framework to unlabeled nodes.

The locations of genes in genomes are often specific, and the gene synteny network extracted from the same genus could reflect the intrinsic properties of the genus. The adjacency probability matrix is defined as the probability that two specific nodes can achieve a certain number of connections in a specific genome synteny network. Adding the adjacency probability matrix to the input was found to greatly improve the performance of the framework (Figure 5B). The adjacency probability matrix provides PPA-GCN with an information dissemination pattern for a specific bacterial genus in the gene synteny network.

Since the adjacency probability matrix can be used to extract synteny information patterns for specific microbial species, we wished to verify whether the gene synteny network could be replaced. Two types of random networks were designed while keeping the degree distribution constant. In one case, the arrangement of the gene positions in each sample genome was disrupted, and in the other, the positional relationships of all genomes were disrupted. Three strategies were considered for feature selection: not including any adjacency probability matrix, including the adjacency probability matrix of the newly generated network, and including the adjacency probability matrix of the real network. The training results show that (Figure 5C), regardless of which random network was used, the training performance when using a random network was much lower than that achieved using the real network. Interestingly, the true probability adjacency matrix can improve the framework training performance, while including the matrix of a random network actually impairs performance. This further shows that the adjacency probability matrix can capture specific information patterns of bacterial genomes. The gene synteny network and the adjacency probability matrix can provide the framework with different information patterns, and neither can replace the other.

### Effectiveness of Self-Supervised Learning Inspiration

**FIGURE 6:**
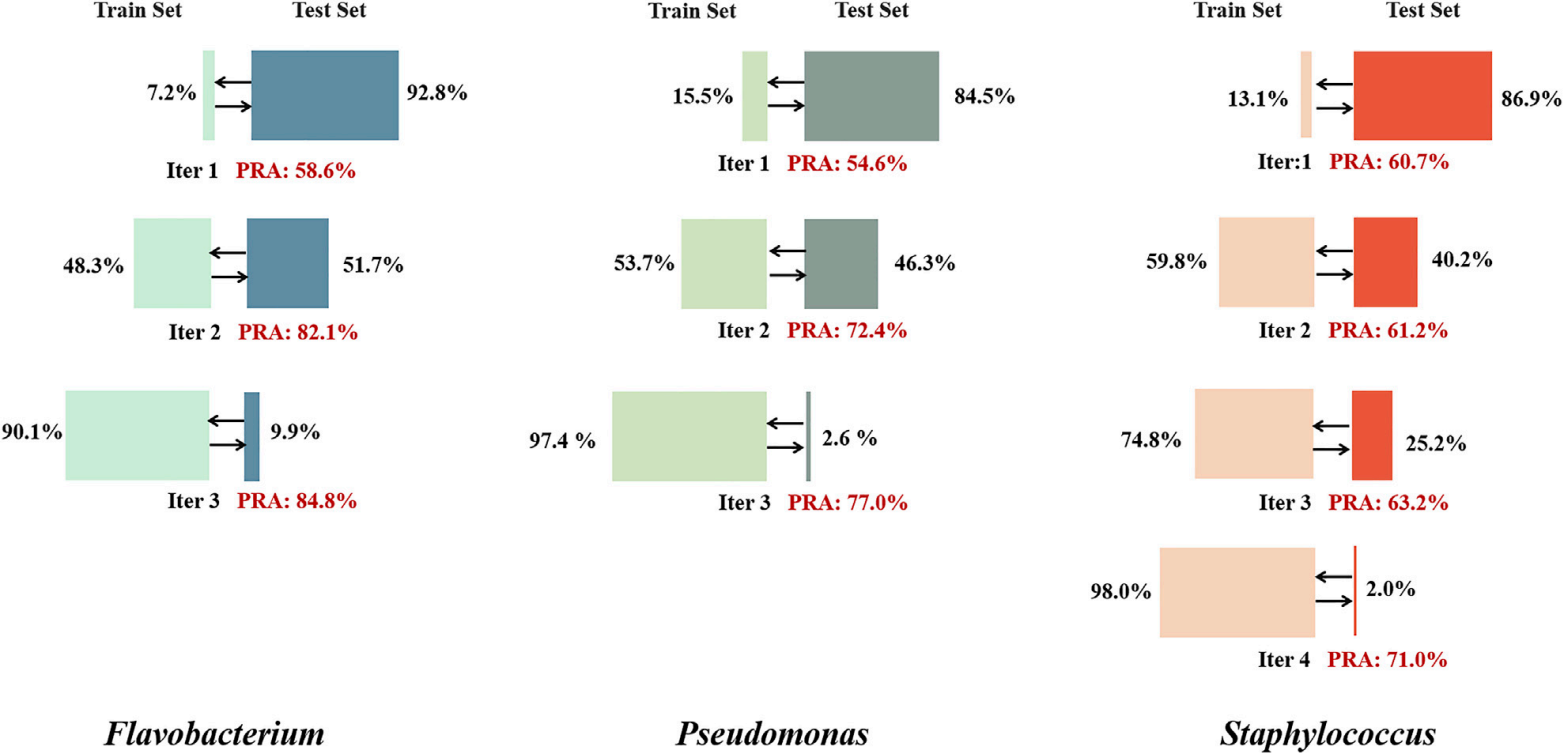
Self-supervised learning iteration results for the three genera. In each iteration, training was performed for 50 epochs, nodes with an average PRA of less than 30% were removed from the training set, and nodes with stably assigned labels in the test set (with assignment consistency over more than 90% of epochs) were added to the training set. When the proportion of the total number of nodes included in the training set exceeded 90%, the final results were output.

Currently, the assignment rate for gene metabolism pathways is lower than 50% in the KEGG GENES database. For the tested genera of three prokaryotes, the assignment rate for metabolic pathways is less than 20% of all nodes in the network, which greatly limits the training performance. The inspiration of self-supervised learning was adopted to extend the training set. Nodes with low PRAs in the validation set were temporarily excluded from the training set, and nodes with highly stable assigned labels in the test set were temporarily added to the training set. After several iterations, the performance eventually stabilized and showed a great improvement over the initial performance (Figure 6).

**FIGURE 7:**
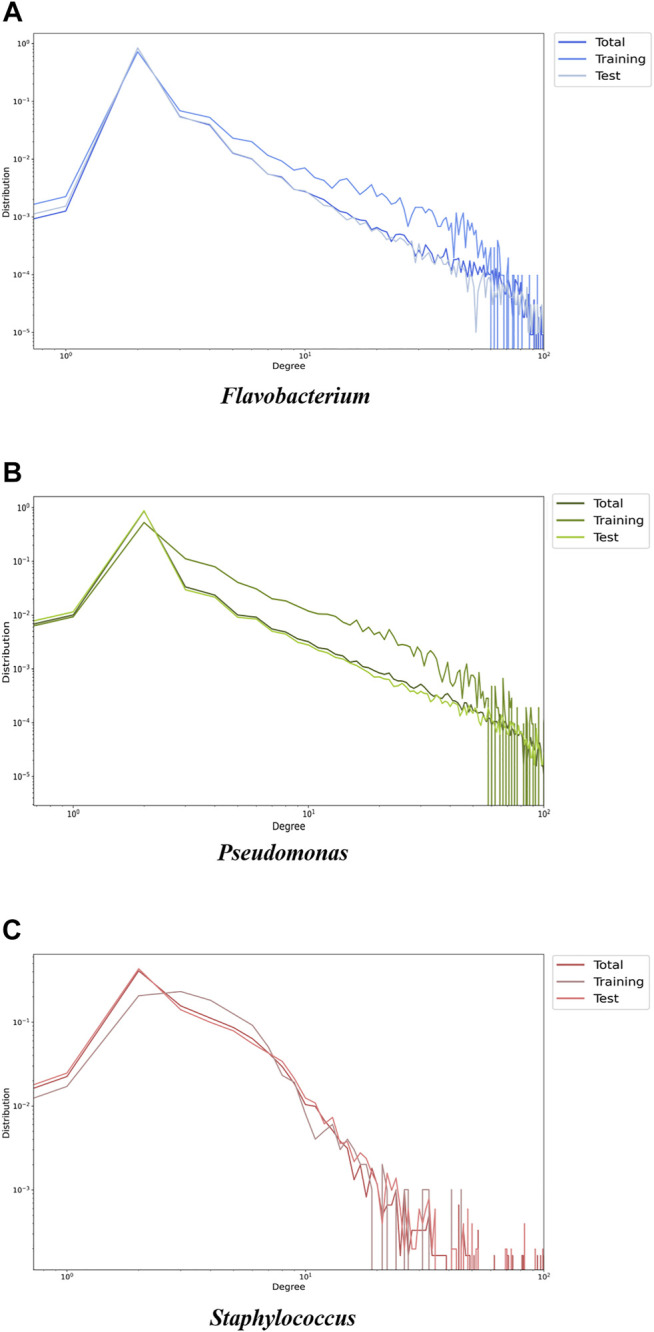
Degree distribution curves describing the degree distributions of the overall network, the training set network and the test set network for each of the three genera [**(A)** *Flavobacterium,* **(B)** *Pseudomonas* **(C)** *Staphylococcus*]. The horizontal axis is the degree (truncated to 100), and the vertical axis is the probability distribution (The sum of the probabilities is 1). The results show that the degree distributions of the initial training set and the test set for the three genera are different, reflecting the genome characteristics of each genus to a certain extent.

We speculate that PPA-GCN’s performance could be significantly improved because labeled nodes spread node attributes in a certain pattern, ultimately causing the entire gene synteny network to present a genus-specific information pattern. The question of whether this kind of propagation can be universally applied to different types of gene synteny networks or is suitable only for network structures with a more “uniform” topology should be considered. Labeled and unlabeled nodes were extracted to construct training and test networks, respectively, and the topological structures of the two new networks were compared. Because PPA-GCN iteratively extracts information from the first- and second-order neighbors of nodes, the tightness of the first- and second-order connections in the network, as measured in terms of the degree distribution and clustering coefficient, need to be considered. The results (Figure 7, Supplementary Table S4) show that the degree distributions of the initial training set and the test set for the three genera are different, reflecting the genome characteristics of each genus to a certain extent. The degree distribution curves and clustering coefficients for the closed pan-genome (*Staphylococcus*) are not significantly different between the initial training set and the test set; in contrast, the initial training set networks of the open pan-genomes (*Flavobacterium* and *Pseudomonas*) are more closely connected than the test set networks, and the overall networks exhibit some level of inhomogeneity. These findings show that the self-supervised inspiration can effectively adapt to gene synteny networks with different topologies.

### The Impact of Different Types of Genomes on Training

Synteny has been used to filter, organize and process local similarities between genome sequences of related organisms to build a coherent global chromosomal context (Deb et al., 2020). Each genus of prokaryotes possesses characteristic genomic gene synteny information, and its patterns are broadly associated with many bacterial functional traits (Brbić et al., 2016). Integrating gene synteny data from one genus can provide assistance to the functional pathway assignments of all genes.

Whether different types of genomes would affect training results should be considered. In addition to the node scale, the run number of self-supervised iterations needed to reach convergence can also reflect differences between different types of genomes. *Staphylococcus* requires more iterations to reach a steady state than *Flavobacterium* or *Pseudomonas*. This suggests that the information pattern of a closed pan-genome is relatively conservative and cannot be easily extended, while the information pattern of an open pan-genome is easier to spread. PPA-GCN could provide insights for judging genome types in accordance with the number of iterations needed for self-supervised learning when analyzing the genome of an unknown species.

### The Role of Hyperlink Nodes in the Gene Synteny Network

There are several nodes with a “super connection number” in the gene synteny network of each genus. Further analysis revealed that these hyperlinked nodes have certain similarities in function. A large proportion of such nodes is assigned to mobile genetic elements (MGEs), which have the potential to disrupt the synteny of the involved genomes and are considered to cause gradual changes (sometimes mutations) in biological genes and promote biological evolution (Muszewska et al., 2019; Richards et al., 2019).

**FIGURE 8:**
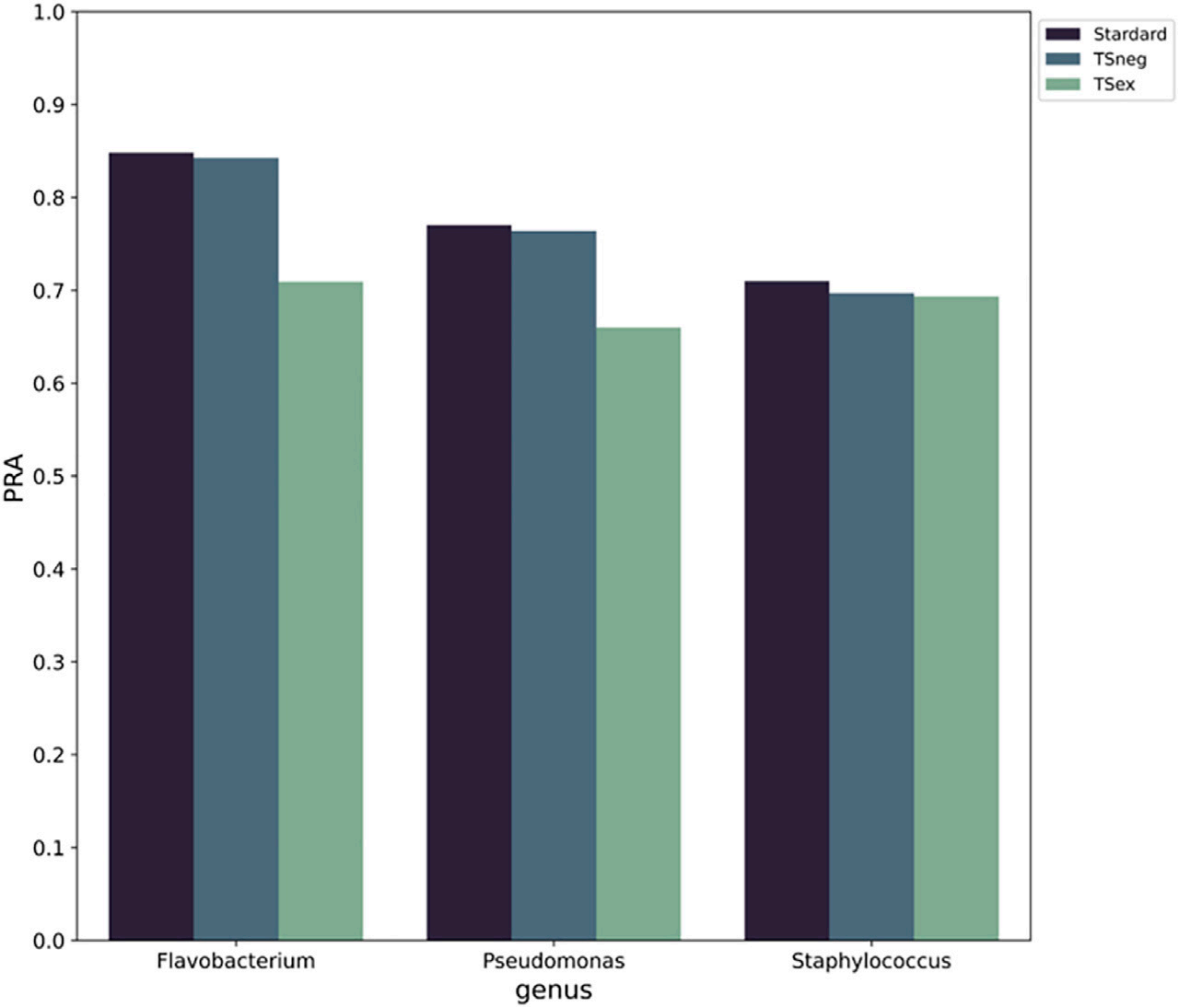
The impact of MGEs on PPA-GCN performance. The horizontal axis represents standard training for the three genera (left), training with the MGEs as negative samples (middle) and training with the MGEs removed from the gene synteny network (right). The vertical axis uses PRA as an evaluation index. The results show that when the MGE nodes are removed from the networks, the performance of PPA-GCN is significantly reduced. When they are used as negative samples, the performance of the framework is only slightly reduced.

We investigated whether the insertion of MGEs into the genomes is random and has an impact on the pattern of functional labels. Two sets of experiments were designed. In the first set, all MGE nodes were removed from the gene synteny network to verify whether the insertion of the MGEs disrupted the information pattern of the original gene synteny networks. In the second set, all MGE nodes were added to the training set as negative samples to verify whether the intervention of the MGEs affected the distribution of functional labels. The results show (Figure 8) that when the MGE nodes are removed from the networks, the performance of PPA-GCN is significantly reduced. When they are used as negative samples, the performance of the framework is only slightly reduced. This indicates that from the perspective of gene location, MGEs may constitute an important part of the gene synteny network of a specific genus, and removing them will destroy the information pattern of the existing gene synteny network. Moreover, MGEs do not interfere with the distribution pattern of gene function.

## Discussion

In present, PPA-GCN is the first deep learning framework that uses genomic structure information to directly assist metabolic pathway assignments of prokaryotic genomes against KEGG information. Datasets representing three genera (*Flavobacterium*, *Pseudomonas and Staphylococcus*) were used to evaluate the assignment rate of the framework, and on all of them, good performance and strong fault tolerance were achieved. These results support the broad application of PPA-GCN to prokaryotic genomic research. For example, it can provide support for the mechanism research of pathogenic bacteria and the design of synthetic biology elements, modules and pathways.

Although all bacterial genome had been fragmented and shuffled by the endless genomic reconstruction and horizontal gene transfer, the localized genome structure was conserved within specific genus of bacteria. Gene synteny structure is intrinsic and stable under genus level and PPA-GCN relies on it. PPA-GCN captures the graph structure and node attributes from the gene synteny information through a graph convolutional network. To maximize the given pathway information of genomes of a genus, PPA-GCN obtains and mines as many possibilities for label assignment through the network as possible. Then PPA-GCN constructs the adjacency probability matrix to evaluate all possibilities, improving the certainty of all assigned labels. The idea of self-supervised learning is adopted to expand the training set and reinforce the training process.

PPA-GCN has the potential for further improvement. The runtime and memory usage of PPA-GCN will be optimized (Supplementary Table S5). At present, only one kind of graph information (the gene synteny network) is used to make assignments. In the future, some other information networks could be incorporated to improve the performance of PPA-GCN, potentially providing the perfect complement to the existing framework, such as a protein-protein interaction network and gene co-expression network.

PPA-GCN exhibits good performance and shows promise to help guide experimental verification and provide considerable additional space for downstream analysis. PPA-GCN could be applied to more genera of prokaryotes with sufficient whole genome sequences and used to build a database of consensus sequences from the perspective of functional pathway assignment, that could describe the differences in prokaryotes of various genera. In short, we present a deep learning framework with great potential to explain the relationship between gene synteny and KEGG pathway information in prokaryotes, which can provide novel insights into functional pathways assignments and is likely to inform future deep learning applications for interpreting functional annotations.

## Data Availability

The original contributions presented in the study are included in the article/Supplementary Material, further inquiries can be directed to the corresponding authors.
