# Supplementary material for: PPA-GCN: A Efficient GCN Framework for Prokaryotic Pathways Assignment
Source: Front Genet. 2022 Apr 4;13:839453. doi: 10.3389/fgene.2022.839453 (PMC9013948; doi:10.3389/fgene.2022.839453)
Supplement: Supplementary file 1 [file DataSheet1.docx]

**Supplementary data**

**Supplementary Table S1: Statistics of the original data for the three genera**

| **Genus** | ***Flavobacterium*** | ***Pseudomonas*** | ***Staphylococcus*** |
| --- | --- | --- | --- |
| Number of samples | 65 | 100 | 500 |
| Total number of proteins | 243834 | 550752 | 1332382 |
| Number of assigned proteins | 67539 | 272388 | 400478 |
| Proportion of assigned proteins | 27.7% | 49.5% | 30.1% |
| Number of nodes | 51247 | 79941 | 10074 |
| Number of integrated nodes | 16876 | 30324 | 5757 |
| Number of single nodes | 34371 | 49617 | 4317 |
| Number of assigned nodes | 3694 | 12429 | 1324 |
| Proportion of assigned nodes | 7.2% | 15.5% | 13.1% |

Assigned proteins refer to proteins that have been assigned functional pathways.

Assigned nodes refer to the node that contains proteins that have been assigned functional pathways.

Integrated nodes refer to the node contains more than one protein.

single nodes refer to the node contains only one protein.

**Supplementary Table S2: Statistics of the single error results for the three genera**

| **Genus** | Add 5% error label | Add 10% error label | Add 15% error label | Add 20% error label |
| --- | --- | --- | --- | --- |
| ***Flavobacterium*** | 99.2% | 97.1% | 96.5% | 93.1% |
| ***Pseudomonas*** | 96.7% | 96.0% | 94.4% | 91.3% |
| ***Staphylococcus*** | 99.7% | 99.3% | 98.7% | 92.8% |

**Supplementary Table S3: Results of feature importance tests**

| **Genus** | ***Flavobacterium*** | ***Pseudomonas*** | ***Staphylococcus*** |
| --- | --- | --- | --- |
| Standard | 0.848 | 0.770 | 0.710 |
| No scale | 0.819 | 0.770 | 0.700 |
| No probability | 0.507 | 0.517 | 0.518 |
| Random with true pro | 0.504 | 0.551 | 0.418 |
| Random without pro | 0.466 | 0.465 | 0.407 |
| Random with pro | 0.465 | 0.423 | 0.389 |
| Partly random with true pro | 0.558 | 0.548 | 0.410 |
| Partly random without pro | 0.480 | 0.466 | 0.402 |
| Partly random with pro | 0.467 | 0.436 | 0.402 |

**Supplementary Table S4: Results of topological analysis**

| **Genus** | ***Flavobacterium*** | ***Pseudomonas*** | ***Staphylococcus*** |
| --- | --- | --- | --- |
| Overall average degree | 2.89 | 3.42 | 4.03 |
| Average degree of training set | 6.67 | 4.56 | 6.48 |
| Average degree of test set | 3.38 | 3.42 | 4.92 |
| Overall Clustering coefficient | 0.11 | 0.13 | 0.36 |
| The clustering coefficient of the training set | 0.15 | 0.16 | 0.38 |
| The clustering coefficient of the test set | 0.12 | 0.13 | 0.37 |

**Supplementary Table S5: Test of execution time and memory usage**

| **The number of genomes** | **Execution time (mins)** | | **Peak memory usage (MiB)** | |
| --- | --- | --- | --- | --- |
|  | **Construction** | **Training** | **Construction** | **Training** |
| **50** | 103.2 | 230.9 | 799.5 | 696.9 |
| **100** | 134.7 | 334.7 | 1037.8 | 742.5 |
| **200** | 220.4 | 642.0 | 1584.6 | 770.3 |
| **300** | 280.9 | 661.4 | 2312.3 | 821.1 |
| **500** | 413.5 | 785.8 | 3332.4 | 922.1 |

PPA-GCN is divided into two steps: network construction and model training.

The test environment: the system version is centos 6.9, the memory is 1.5T, and the CPU is 60 threads.

The test genus is *Staphylococcus*
